# Blind spots in artificial intelligence systems: poor identification of self-generated medical images–evidence-based cross-sectional study

**DOI:** 10.3389/fmed.2026.1895176

**Published:** 2026-08-11

**Authors:** Sultan Ayoub Meo, Muhammad F. Mubarak, Riham A. ElToukhy

**Affiliations:** 1 Department of Physiology, College of Medicine, King Saud University, Riyadh, Saudi Arabia; 2 Department of Medicine (Gastroenterology Unit), College of Medicine, King Saud University, Riyadh, Saudi Arabia; 3 Department of Family and Community Medicine, College of Medicine, King Saud University, Riyadh, Saudi Arabia

**Keywords:** artificial intelligence, blind spot, ChatGPT, cognitive dysfunction, Google Gemini

## Abstract

**Objectives:**

Artificial intelligence (AI) systems are increasingly adopted in scientific visualization, clinical sciences, medical education, and health sciences research. Despite its impressive generative capabilities, the AI role remains poorly established in some areas. This study aims to investigate whether AI systems, ChatGPT-4 and Google Gemini 1.5 Pro, can identify their own generated images.

**Methods:**

In this study, AI systems, OpenAI’s ChatGPT-4 and Google Gemini 1.5 Pro, were employed to generate and identify medical science images. AI-generated images in medical science span 16 clinical illustrations: 8 anatomical and 8 pathophysiological mechanisms. The images were subsequently reintroduced into the same system for identification. The descriptive statistics, including numbers, percentages, and accuracy rates, across the images were analyzed. For a correct answer or an incorrect answer, a score of 1 was allocated; a *p*-value less than 0.05 was considered significant.

**Results:**

The results revealed that Artificial Intelligence models, ChatGPT and Google Gemini, were significantly less able to identify AI-generated images correctly. There were significantly higher incorrect rates than correct identification rates for AI-generated images overall (27/32 incorrect vs. 5/32 correct; 84.37% vs. 15.62%; *p* = 0.001). ChatGPT showed an overall accuracy of 18.75% (*p* = 0.021), while Google Gemini demonstrated an overall accuracy of 12.5% (*p* = 0.004). The poorest performance was observed in Google Gemini anatomy images, with no correct identifications (0%, *p* = 0.008).

**Conclusion:**

Artificial Intelligence models, ChatGPT and Google Gemini, have limited ability to identify AI-generated images correctly. The results demonstrate structured, architecture-dependent patterns of image identification dysfunction. The study emphasizes implications for AI governance and validation protocols in research, medicine, and medical education.

## Introduction

Artificial intelligence (AI) has evolved from a theoretical concept to a useful instrument integrated into contemporary scientific and medical processes (1). Large language models (LLMs), such as ChatGPT-4 and Google Gemini, have demonstrated tremendous potential and disruptive influence in recent years. They have advanced quickly and performed exceptionally well across various disciplines. The medical sciences are unquestionably one of the most promising sectors for the use of AI because of their enormous volumes of data and intricate procedures. LLMs are increasingly being incorporated into clinical practice, medical research, and medical education. However, AI still faces challenges in real-world applications, including ethical dilemmas, interpretability issues, and hallucinations (2).

The multimodal architecture has enabled systems to generate coherent text, images, diagrams, and analytical interpretations across highly specialized domains. In medical sciences, AI-generated images are increasingly used for education, research and clinical practice (2). However, as AI systems become more sophisticated, questions arise regarding their epistemic boundaries (3) and self-recognition. Can an AI system identify its own generated image? In human cognition, self-awareness and metacognition allow individuals to reflect on their actions, distinguish self-generated content from external content, and evaluate the quality and origin of their outputs (4).

Artificial intelligence systems operate on statistical pattern recognition without intrinsic awareness (5). AI generates output based on learned distributions rather than self-directed cognition. This raises an important conceptual gap: while AI can produce content indistinguishable from human-created material, it may lack the ability to recognize its own contributions once produced. Therefore, this study aimed to empirically explore this gap by evaluating whether an AI system can correctly identify its own generated images. The findings can contribute to ongoing discourse in AI interpretability, reliability, and the philosophical boundaries of machine cognition.

## Research methodology

### Study design and settings

This cross-sectional analytical study was conducted in the Department of Physiology, College of Medicine, King Saud University, Riyadh, Saudi Arabia, from March 1, 2026, to May 1, 2026. This study evaluates the cognitive knowledge of the large language models ChatGPT-4 and Google Gemini 1.5 Pro for AI-generated image identification. The study was conducted in two phases: image generation and image identification.

### Phase 1: image generation

In this study, a total of 32 images were generated using an AI system, a large-language model, 16 images by ChatGPT-4 and 16 images by Google Gemini 1.5 Pro, to evaluate the AI models’ cognitive knowledge in self-generated image identification. For image generation, specific keywords or short sentences with clear instructions were entered into ChatGPT and Google Gemini, and a command to generate the image was issued. The individual images were created, downloaded, and a serial number was allocated and stored. After image generation, the images were deleted from the AI systems ChatGPT-4 and Google Gemini to minimize recall bias.

Any blurred image, labeling indicating the image is AI-made, visible watermarks, text or labeling, prompts, or visual clues such as “AI Generated” or highly congested labeling were excluded from the study. The anatomical images were consistent in structures, and the pathophysiological images were consistent in etiopathological mechanisms. All images were colored, clear, of appropriate resolution, and easy to understand. To maintain the originality of the images, we did not perform any major adjustments, modifications, or resizing. However, main headings or unnecessary labels were deleted. The research team member with expertise in basic and clinical medical sciences checked the accuracy of all the images before submitting them for identification.

Medical images generated and identified by ChatGPT-4: A total of 16 medical science images span clinical illustrations, including anatomical (8 images) and pathophysiological mechanisms (8 images). The 08 human Anatomy images generated and identified by ChatGPT-4 include neurons, heart, lungs, kidneys, liver, pancreas, gallbladder and spleen. The pathophysiological figures (08) include: diabetes mellitus; air pollution and diabetes; risk factors for the development of diabetes mellitus; genetics, environmental, lifestyle, and type 1 diabetes mellitus; inflammatory response and type 1 diabetes; air pollution and gestational diabetes; air pollution and Alzheimer’s disease; cell membrane organelles.

Medical images generated and identified by Google Gemini images: The human Anatomy images (08) generated and identified by Google Gemini Pro include liver, gallbladder, heart, thyroid, parathyroid, neuron, trachea, and cones. The pathophysiological figures (08) include oxidative stress, air pollution and hypertension, air pollution and myocardial infarction, lifestyle and diabetes, lung and air pollution, air pollution entry into the blood, air Pollution and COPD, and air Pollution and atherosclerosis. Each model evaluated only the images it had generated itself.

**FIGURE 1:**
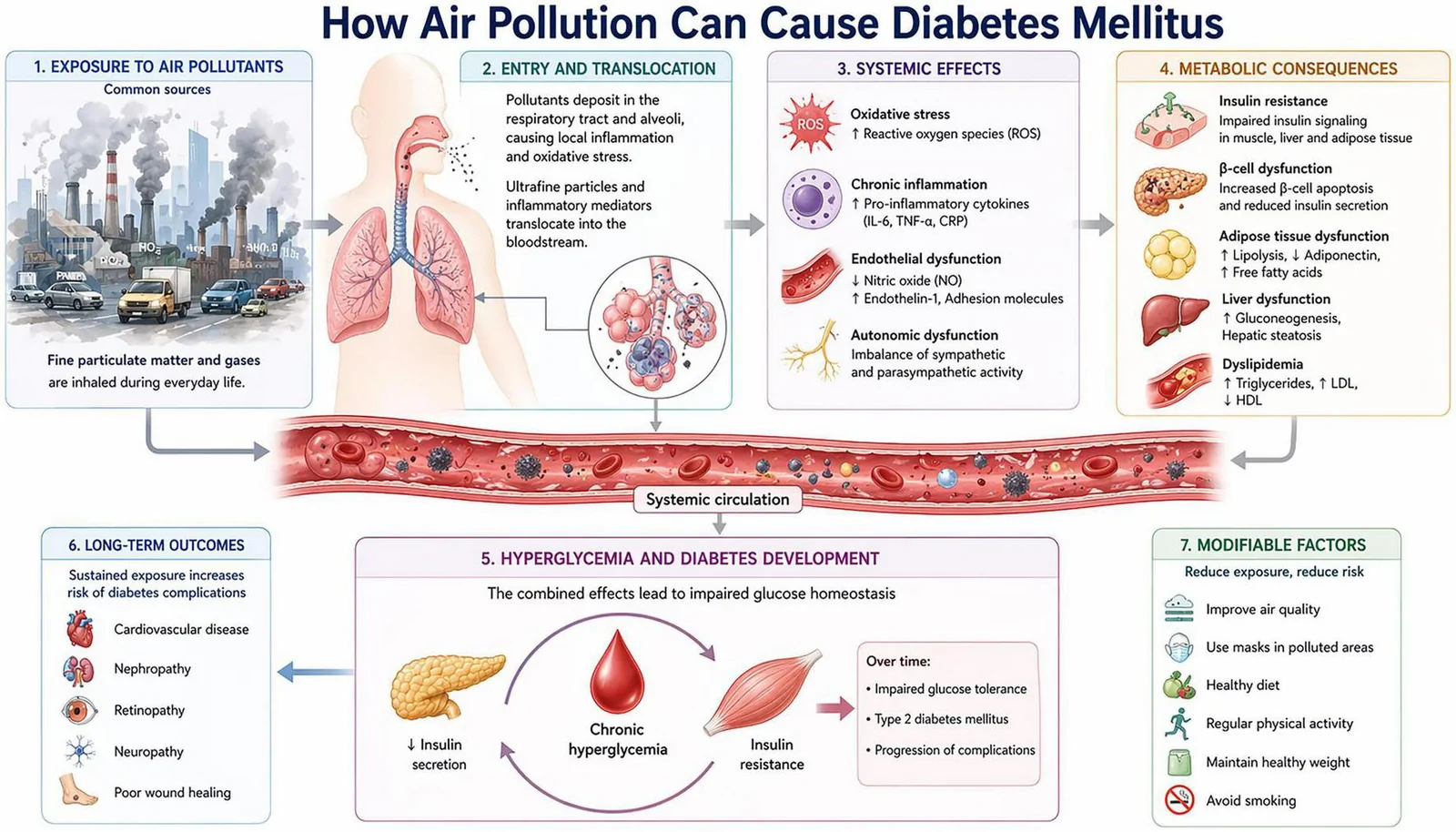
Artificial intelligence-generated medical image and identification.

Each image was generated using structures that emphasize clarity, labeling, and an academic-style presentation (Figure 1). Each image was numbered with a specific identification serial number and classification as an anatomical or pathological image. These 16 images were reintroduced individually into the same AI system, ChatGPT-4 and Google Gemini, for their identification, whether the image is AI-generated or human-generated, without access to metadata or prior context information.

### Phase 2: image identification

After a cooling interval to avoid immediate contextual association, each figure was presented individually to the AI systems. The images were entered one at a time, and a new session was generated for each entry to minimize the risk of memory bias. ChatGPT and Google Gemini self-generated image identification knowledge in each image identification was investigated. The system was prompted as follows: “Identify whether the following figure is AI-generated or human-made (Figure 1). The “regenerate response” was not used; instead, we considered the original response as the final one. The responses were in “yes or no, or unable to identify, or most likely not AI-made.” For a correct answer or an incorrect answer, a score of 01 was allocated. The responses were calculated, and each correct or incorrect response was counted.

### Ethical considerations and statistical analysis

In this study, no human subjects were involved; therefore, IRB approval was not required under institutional policy. The AI models, ChatGPT and Google Gemini, were used for image generation and identification; no model was fine-tuned or altered during the study. Descriptive statistics, including numbers, percentages, and accuracy across the images, were recorded. All responses were entered into a computer and analyzed using SPSS statistics version 22. Responses were classified as either correct or incorrect. For each correct or incorrect response, a score of 01 was assigned. All scores were calculated and tabulated. An exact binomial test was conducted to assess the significance of the observed response proportions, and *p*-values were calculated. Furthermore, Cohen’s h was calculated to estimate the effect size between the groups. A *p*-value of less than 0.05 was considered significant.

## Results

**FIGURE 2:**
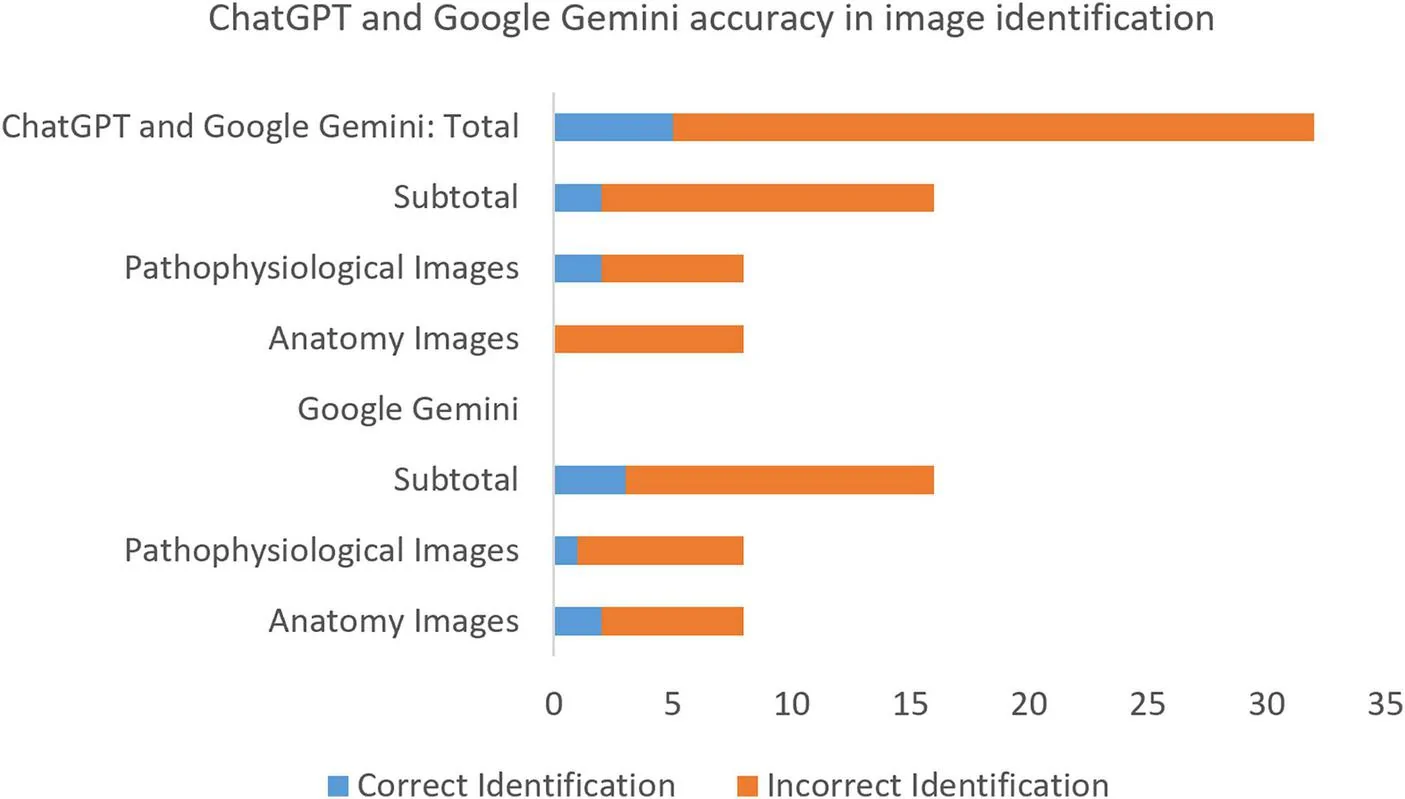
ChatGPT and Google Gemini responses in medical image identification.

Artificial intelligence (AI) systems increasingly exhibit complex, cognition-like behaviors, yet systematic frameworks for evaluating their dysfunction remain underdeveloped. Here, we present a cross-sectional study of AI systems spanning large language models. While analyzing the data, the results revealed that Artificial Intelligence Large Language Models, ChatGPT-4 and Google Gemini 1.5 Pro, both showed substantially more incorrect answers than correct identifications of AI-generated images. A total of 16 anatomical and pathophysiological images were manually entered, one at a time. Assignments were given to ChatGPT and Google Gemini to identify whether each image was AI-generated or human-made. The overall performance for both ChatGPT and Google Gemini was statistically significant (*p* = 0.001), indicating that AI systems were significantly less able to correctly identify AI-generated images (Figure 2 and Table 1).

**TABLE 1:** Artificial intelligence systems, ChatGPT and Google Gemini accuracy in medical sciences image identification.

Image type	Correct identification	Incorrect identification	Accuracy (%)	Significance level
**AI-generated images**				
ChatGPT-4 (n = 16)				
Anatomy images	2/8 (25.0%)	6/8 (75%)	25.0 %	*p* = 0.289
Pathophysiology images	1/8 (12.5%)	7/8 (87.5%)	12.5%	*p* = 0.070
Subtotal	3/16 (18.75%)	13/16 (81.25%)	18.75%	*p* = 0.021
Google Gemini 1.5 Pro (n = 16)				
Anatomy images	0/8 (0%)	8/8 (100%)	0.0%	*p* = 0.008
Pathophysiology images	2/8 (25.0%)	6/8 (75%)	25.0%	*p* = 0.280
Subtotal	2/16 (12.5%)	14/16 (87.5%)	12.5%	*p* = 0.004
Total: both AI models	5/32 (15.62%)	27/32 (84.37%)	15.62%	*p* = 0.0001

Google Gemini showed the poor performance when identifying anatomy images (0% accuracy; *p* = 0.008) compared with ChatGPT. Exact binomial testing demonstrated significantly higher incorrect than correct identification rates for AI-generated images across both ChatGPT and Google Gemini (27/32 incorrect vs. 5/32 correct; 84.37% vs. 15.62%; *p* = 0.001). ChatGPT showed an overall accuracy of 18.75% (*p* = 0.021), while Google Gemini demonstrated an overall accuracy of 12.5% (*p* = 0.004). The poorest performance was observed in Google Gemini anatomy images, with no correct identifications (0%, *p* = 0.008) (Table 1).

ChatGPT-4 achieved overall accuracy marginally higher than that of Google Gemini. ChatGPT-4 correctly identified 3 out of 16 images (18.75%); however, Gemini correctly identified 2 out of 16 images (12.5%). The effect size, as measured by Cohen’s h, was 0.17, indicating a very small, non-significant difference between the two AI models. It showed that both AI models demonstrate nearly equal performance in image identification, with a very small difference in effect size. There was a similar trend across both AI models, with exceptionally low accuracy in identifying AI-generated images. Further, the results reveal that AI did not correctly identify its own generated images. It is like creating it but not recognizing it as your own.

## Discussion

Artificial intelligence has accelerated advancements in research, technology, healthcare environments, and medical education in recent years (6)–the AI integration in healthcare systems offers transformative opportunities (7). The literature highlights AI’s role in medical laboratories, including hematology, biochemistry, pathology and radiology. AI can analyze huge and complex data, including electronic health records, medical imaging, and genomic profiles, to recognize trends, patterns, predict disease progression, and optimize management strategies (8, 9). However, the literature also highlights concerns about the use of AI in health sciences. The present study investigates whether AI systems, ChatGPT and Google Gemini, can identify their own-generated images. In the present study, it was observed that AI models, ChatGPT and Google Gemini, have less potential to correctly identify AI-generated images (Figure 2 and Table 1). This phenomenon could be explained as cognitive dysfunction or blind spots in a biological sense, as the system lacks consciousness and understanding while identifying its own AI-generated images.

Human cognition includes metacognitive processes, monitoring and evaluating thoughts and actions (10). The AI system evaluated in this study lacks internal models for identifying self-generated content. This lack of image identification knowledge explains the complete inability to recognize the image outputs and raises questions about its validity and reliability. The identification of AI-generated content (AIGC) is increasingly recognized as a significant challenge for various reasons, including trust, content credibility, and academic reliability (11, 12).

Artificial intelligence has a promising association with the healthcare sector and has achieved widespread global adoption. Its integration into healthcare delivery enhances diagnostic precision, treatment efficacy, and overall public healthcare (13, 14). Meo et al. (6) conducted a study to investigate knowledge of “ChatGPT-4 and Google Gemini 1.5 Pro” in a Multiple-Choice Questions (MCQs) examination and assessed the validity and reliability of these AI tools. Both ChatGPT-4 and Google Gemini 1.5 Pro achieved excellent scores, over 80%, in various medical subjects. In another study, Meo et al. (15) found that Google Bard scored adequately in radiological, histopathological, and morphological image identification and interpretation. The figures and images were obtained from various web sources, medical books, journals, and examination pools. However, in the present study, both ChatGPT-4 and Google Gemini did not correctly identify their own generated images, whether AI-generated or human-made. This is the main concern that AI failed to identify its own generated images.

Duggan et al. (16) reported that AI produced comprehensive and visually engaging images; however, it failed to portray perfect anatomy and often included predicted structures. The authors suggested that more research is required to train and fine-tune AI models to produce accurate and appropriate images in the health sciences. The medical imaging machine learning research is a new paradigm for traditional radiology and health sciences investigators. There are new procedures, limitations, and challenges that must be managed when performing state-of-the-art data science analysis and image identification (17).

Artificial intelligence has made remarkable progress in image recognition (18–22). However, incorporation of AI in radiological practice requires intensified monitoring of its utility and safety. The collaboration between physicians, developers, and regulators enables further monitoring of AI performance and the addressing of ethical issues. AI fulfills its promise to advance patient well-being if all steps, from development to integration into healthcare sciences, are thoroughly evaluated. In radiology, image processing algorithms still need broad modifications. Today, the outcomes of AI in image processing cannot be fully trusted. In other areas of medicine, rather than image processing, this implantation problem remains a challenge (23, 24).

In recent years, the scientific literature has highlighted the significance and limitations of AI tools for generating images in clinical sciences and medical education. Kumar et al. (25) investigated the potential limitations of text-to-image tools in generating images for medical education. They focused on illustrations of vital physical signs on the human face and raised concerns about photographs. The literature highlights that AI can generate images from text for research, education, and communication. However, these images may undermine trust and lead to misrepresentation (26, 27).

Similarly, Paslı et al. (28) investigated the strengths and limitations of AI image generation in anatomy education and found a notable lack of anatomical features and visual quality in the generated images. Eldesoqui et al. (29) assessed and compared the anatomical accuracy of graphics produced by four AI text-to-image generators, including Microsoft Bing, DeepAI, Freepik, and Gemini, emphasizing their value in medical education. This study examined the potential of generative AI for medical illustration and identified significant limitations in accuracy and details, especially in bony structures. Although AI accelerates the drawing process, it cannot replace the proficiency of skilled medical illustrators. These findings also support our study findings.

**FIGURE 3:**
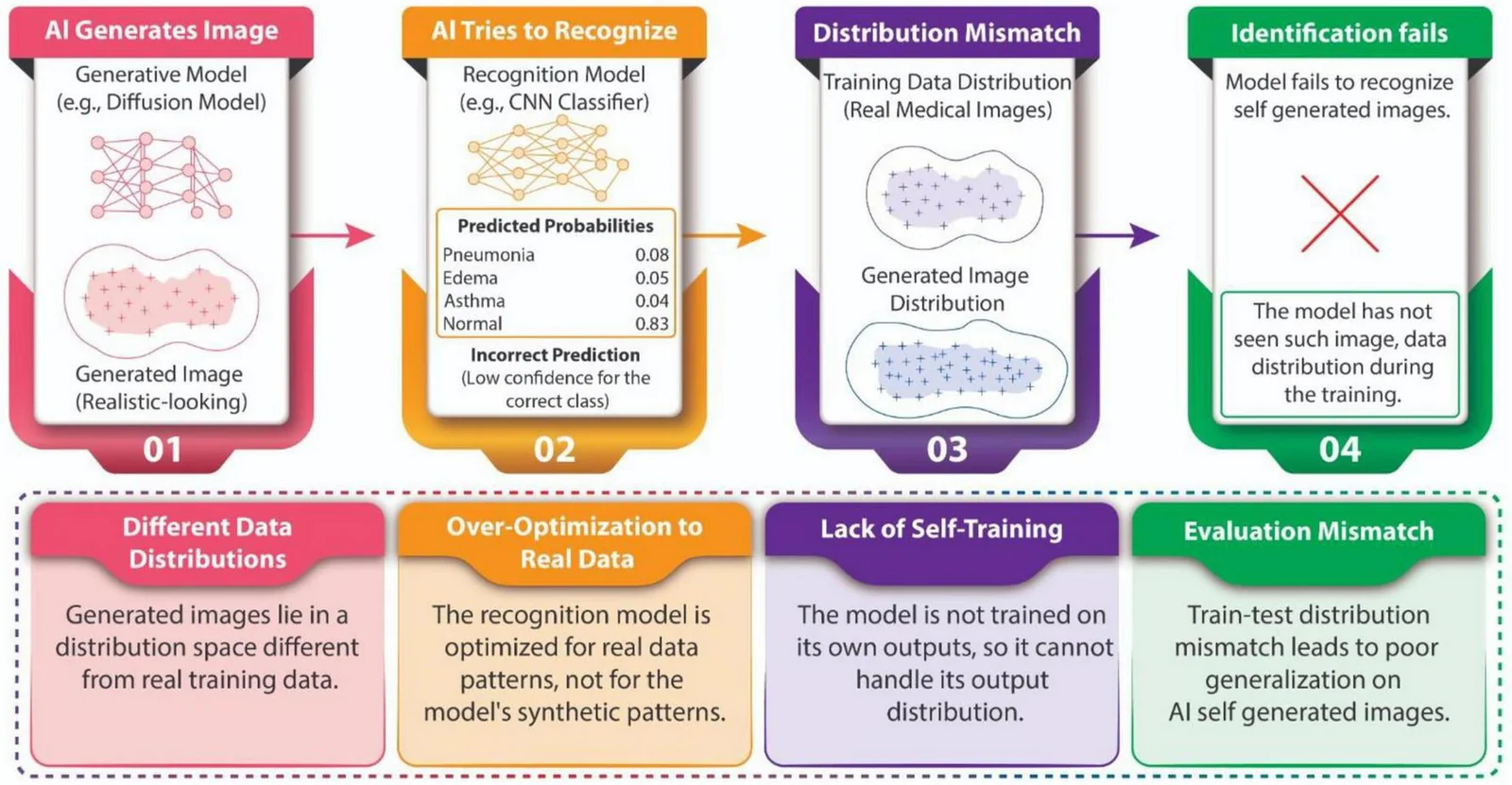
The potential mechanisms by which AI is unable to identify its own generated image.

In our study, it is vital to understand why the AI is unable to identify its own generated images. AI systems are unable to recognize their own-generated medical images due to technical and domain-specific constraints, since generative AI models and AI detectors are developed independently. In contrast to natural images used to train AI detectors, medical images have standardized anatomical features, grayscale patterns, and repeating textures. AI detection faces multiple challenges, as contemporary diffusion and GAN-based models can generate extremely realistic medical images with precise anatomical characteristics. The majority of AI models and detectors have not seen such data and are not trained, which results in poor generalization and identification in medical imaging (Figure 3). The AI models frequently exhibit significant false-negative rates, particularly after image alterations such as compression, resizing, and annotation.

Furthermore, another aspect of this limitation is that, in AI systems, each query is processed independently unless memory systems are explicitly integrated. As a result, the system cannot inherently connect generation events (image creation) and evaluation events (identification task). This architectural separation produces epistemic discontinuity. The AI relies on learned data between visual features and training labels. In datasets, high-quality health sciences images are human-generated. The system internalizes a biased relationship, especially toward smart and refined figures; these empirical overrides any consideration of AI-generated images. The findings raise concerns about AI cognition: how it is possible for a system that generates content but cannot recognize it. In that situation, accountability can be assigned when internal origin tracing fails. It shows that the observed “blind spot” is not a defect but a structural issue of non-conscious generative systems (Figure 3).

Moreover, AI may fail to recognize its own-generated medical images because it lacks memory of past outputs and relies solely on pattern recognition. If both the generator and classifier are trained on similar medical datasets, synthetic images can closely mimic real ones, falling inside the classifier’s “real” decision boundary. Additionally, medical images have low structural variability, and advanced generators replicate human-like imperfections. Without exposure to the specific generative model during training, the classifier cannot distinguish self-generated from human-made images, leading to incorrect identification (Figure 3).

## Study strengths and limitations

This study has strengths and limitations. To our knowledge, research on self-generated medical images and their recognition by AI models remains limited in the current scientific literature. We checked the validity and reliability and used two AI models, ChatGPT and Google Gemini, and both showed comparable results. This study has some limitations that must be acknowledged: it could be further validated on other AI systems for cross-validation; other AI architectures may behave differently. In this study, images were exclusively AI-generated, both ChatGPT and Google Gemini, without a corresponding set of human-generated images. There was no human comparator group, and the human ability to detect AI-generated images was not assessed. The small sample size (ChatGPT 16; Google Gemini 16) limits generalizability to existing AI architectures. This cross-sectional design captures cognitive dysfunction at one point only; no inference on causality or temporal stability. In the future, further large-scale studies could be conducted using more advanced AI models, cross-validation (with one model generating and another identifying, and vice versa), and a human comparator group to confirm the consistency and generalizability of these findings and to draw more robust conclusions.

## Conclusion

The AI systems, ChatGPT-4 and Google Gemini 1.5 Pro are unable to correctly recognize their own-generated medical images. The limitation arises from architectural constraints, including stateless processing, lack of self-referential modeling, train-test distribution mismatch, and heuristic-based visual interpretation. The study findings underscore the imperative of human supervision in AI-enhanced scientific processes and highlight the need to establish transparent provenance-tracking mechanisms for forthcoming AI implementations.

## Data Availability

The raw data supporting the conclusions of this article will be made available by the authors upon reasonable request.
